# Crohn’s-like disease in a patient exposed to anti-Interleukin-17 blockade (Ixekizumab) for the treatment of chronic plaque psoriasis: a case report

**DOI:** 10.1186/s12876-019-1067-0

**Published:** 2019-09-05

**Authors:** Matthew K. Smith, Jay Pai, Remo Panaccione, Paul Beck, Jose G. Ferraz, Humberto Jijon

**Affiliations:** 1 Department of Medicine, University of Calgary, Calgary, Alberta Canada; 2 Department of Medicine, University of British Columbia, Vancouver, British Columbia Canada; 3 Division of Gastroenterology, University of Calgary, HSC 1667, 2500 University Dr. NW, Calgary, Alberta T2N4N1 Canada

**Keywords:** Inflammatory bowel disease, Crohns disease, Ixekizumab, Anti-IL-17

## Abstract

**Background:**

Plaque psoriasis and inflammatory bowel disease (IBD) are both chronic immune-mediated inflammatory diseases with an overlapping genetic profile and have been linked in epidemiological studies. Psoriasis and IBD share similar components in their inflammatory pathways and animal and human studies have suggested a potential role for targeting interleukin (IL)-17 with novel antibody therapies in the treatment of these diseases. These studies, while promising for psoriasis, have been associated with deterioration in patients with IBD. Post-hoc analyses of clinical trials involving Ixekizumab revealed adverse outcomes in a small cluster of patients with IBD, prompting recommendations to monitor this population with the use of this drug.

**Case presentation:**

Forty-two year old Caucasian male with treatment-refractory chronic plaque psoriasis who developed new onset diarrheal illness and rectal bleeding following a 12 week induction period with Ixekizumab (anti-IL-17 neutralizing antibody). Colonoscopy revealed severe ulceration throughout the ascending and transcending colon. Histopathology, combined with endoscopic findings, led to a diagnosis of Crohn’s-like colitis. The patient’s anti-IL-17 medication was discontinued and endoscopic remission was induced with the use of corticosteroids, escalated anti-TNF therapy and eventually anti IL-12/23 neutralizing antibody (ustekinumab).

**Conclusion:**

Murine studies implicate IL-17 and the downstream effects of its inhibition, in the breakdown of the gut epithelial layer, the disruption of normal host immune responses and the propagation of intestinal inflammation. The increasing use of IL-17 inhibitors has led to reports of exacerbation and potential development of inflammatory bowel disease. While clinical trials have revealed clusters of new inflammatory bowel disease cases amongst psoriasis patients using an IL-17 inhibitor, there remains a lack of evidence to suggest a causal relationship. This is the first case report of de-novo severe Crohn’s-like IBD in association with the use of Ixekizumab requiring rescue with escalated dosing of anti-TNF therapy and highlights the importance of close monitoring in patients being treated with IL-17 inhibitors, especially in those patients with known risk factors for inflammatory bowel disease.

## Background

Plaque psoriasis and inflammatory bowel disease (IBD) are both chronic immune-mediated inflammatory diseases that share an overlapping genetic profile. Crohn’s disease (CD) and Ulcerative Colitis (UC) are the 2 most common forms of IBD [1]. Several epidemiological studies have demonstrated increased CD and UC prevalence among patients with psoriasis [2–4]. Although the exact pathogenesis underlying their co-occurrence is still unknown, psoriasis and IBD share components of their immunopathogic pathways. Animal and human studies have suggested a potential role for interleukin (IL)-17 in these diseases, leading to the targeting of IL-17 using novel antibody therapies [5–7].

Secukinumab, Brodalumab, and Ixekizumab are IL-17 inhibitors that have demonstrated great efficacy in phase 3 clinical trials for the treatment of moderate to severe plaque psoriasis, and psoriatic arthritis [8–10]. However, in clinical trials of CD, both Secukinumab and Brodalumab failed to show any benefit, and have been associated with worsening of CD compared to placebo [11, 12]. This year, a small cluster of IBD cases was highlighted in 2 post-hoc analyses of 8 clinical trials for psoriasis patients exposed to Ixekizumab [13, 14]. Although the rates of CD and UC were relatively uncommon (< 1%), these findings have prompted recommendations to monitor this specific population for new onset or exacerbation of IBD during treatment with Ixekizumab.

We report a new case of colitis with features resembling Crohn’s disease in a patient recently exposed to Ixekizumab for treatment of chronic plaque psoriasis. We appraise the clinical and laboratorial findings, discuss treatment and review the available literature.

## Case presentation

**Fig. 1:**
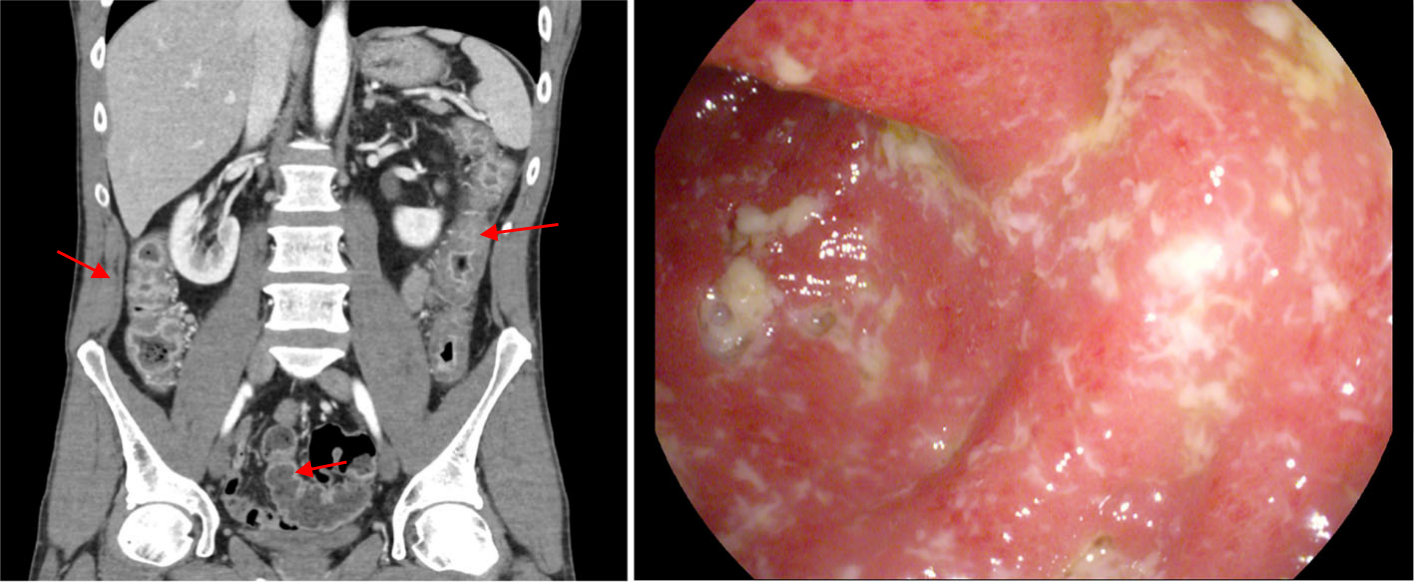
Abdominal CT showing mural thickening from ascending colon, to distal descending colon, with relative rectal and sigmoid sparing (Left). Arrows clockwise highlight descending colon, rectum and ascending colon. Representative image of ascending colon at index colonoscopy (Right)

We report a case of a 42 years-old Caucasian male presenting with 2-weeks of crampy lower abdominal pain, and frequent non-bloody diarrhea associated with urgency, tenesmus, and nocturnal episodes. Additional symptoms included 6 days of subjective fevers, chills and rigors, with no resolution despite regular acetaminophen and ibuprofen. He presented to the emergency department (ED) on day 16 of this episode, where laboratory investigations were significant for an isolated elevated C-reactive protein (CRP) of 280 mg/L [< 8 mg/L]. A CT-scan of his abdomen demonstrated mural thickening of the large bowel from the ascending colon to the distal descending colon, with sparing of the sigmoid colon, as well as reactive retroperitoneal lymphadenopathy (Fig. 1a). The patient remained in the ED with concern over infectious versus drug reaction etiologies, and was discharged home on day 17 with a plan to follow up stool analysis with his family physician. He returned to the ED on day 19 with 24-h of frequent, small volume, rectal bleeding with mucous at which time the Gastroenterology service was consulted. He did not report any nausea or vomiting, arthralgias, oral ulcers, or eye pain. There was no recent travel history, sick contacts, dietary indiscretions, or antibiotic use. There was no past history of gastrointestinal bleeding, or change in bowel habits. His family history was notable for a possible diagnosis of inflammatory bowel disease in his mother. He had a 20 pack-year smoking history, but quit 5 years previous.

The patient’s past medical history was notable for chronic plaque psoriasis, manifesting as palmoplantar disease without joint involvement. Over the past 15 years, flares were managed with 2 to 4-week courses of acitretin and systemic cyclosporine. His treatment was optimized to regular use of these medications over the past year for refractory symptoms. Subsequently, the patient was switched to subcutaneous injections of Ixekizumab, for long term management of his moderate psoriasis. His presenting symptoms manifested approximately 2 days after completing the 12-week induction period.

On physical examination, the patient was hemodynamically stable (Blood Pressure: 135/85 mmHg, Heart Rate: 67 beats/minute) with a low-grade fever (Temperature: 37.5 degrees Celsius). The patient did not look toxic, but was in obvious pain that was exacerbated by truncal movement. Abdominal exam revealed a soft abdomen with tenderness in the left lower quadrant upon palpation, and evidence of rebound tenderness. There were no palpable masses and no hepatosplenomegaly. Digital rectal exam showed a normal external exam, with dark stool, but no bright red blood. Laboratory investigations revealed a drop in hemoglobin from 152 g/L to 134 g/L [130–170 g/L], and a rise in CRP to 291 mg/L [< 8 mg/L] over a 72 h period from the first ED visit. The patient had a very low albumin at 23 g/L [35–50 g/L]. The remainder of blood work, including white blood cell, platelet count, liver enzymes and kidney function were normal. Stool samples for cultures of bacterial pathogens, ova and parasites, viral serologies, *Clostridium difficile* toxin (*C. diff*) and fecal white blood cells were negative. Colonoscopy images were grossly indicative of severe Crohn’s colitis in the cecum, ascending colon and transverse colon, with deep punched-out circumferential ulcers in the transverse and descending colon. There was relative sigmoid and rectal sparing, with noticeable loss of vascular pattern (Fig. 1b). The ileocecal valve was not intubated as it was noted to be very friable and erythematous. Biopsies were negative for viral cytopathic effect and cytomegalovirus (CMV) stain negative, tissue architecture was consistent with severe colitis with rare mucosal granulomas present.

On history and physical examination alone, we considered a differential diagnosis for his abdominal pain, fever and rectal bleeding including infection, inflammatory bowel disease, drug-induced colitis, ischemic colitis, and diverticulitis. These were sequentially ruled out following negative infectious screening tests, abdominal imaging and colonoscopy findings most suggestive of active Crohn’s colitis.

Histopathology from index colonoscopy suggested chronic mild to moderate pancolitis involving the ascending, transverse, descending colons and rectum. Repeat biopsies 2 weeks later showed severe pancolitis with no viral cytopathic effect with rare granulomas. After careful consideration of the entire clinical picture, a tentative diagnosis of Crohn’s colitis was made.

Immediately post index colonoscopy and once infectious causes were ruled out, the patient was started on intravenous steroids (Solumedrol 40 mg IV, daily). Over the next 24 h, the patient remained afebrile and hemodynamically stable. Symptomatically, he reported minimal rectal bleeding and abdominal pain, and was able to tolerate a full fluid diet. His hemoglobin continued to decline (115 g/L), however his CRP trended down slightly to 236 mg/L and with some improvement in his albumin. The patient remained in hospital, with a partial response to steroids demonstrated by his improved clinical status, CRP and albumin.

**Fig. 2:**
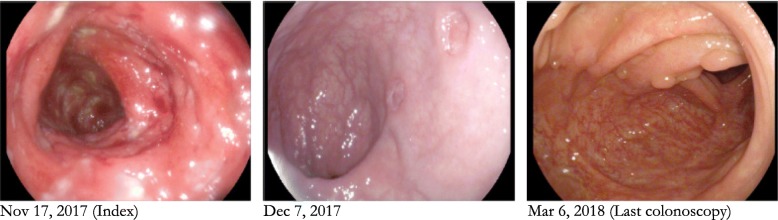
Colonoscopy showing, from left to right, the progression from severe Crohn’s colitis with deep punch out ulcers to healed mucosa in endoscopic remission following corticosteroid and anti-TNF therapy

Ultimately the patient received total parenteral nutrition (TPN) and after 9 days of IV steroids, was induced using an anti-tumor necrosis factor (TNF) neutralizing antibody (infliximab 10 mg/kg) with accelerated dosing 1 week later. Clinical and endoscopic improvement in his colitis was evident on endoscopy 4 days after his second infusion (Fig. 2). Histopathology from his third colonoscopy (4 months post initiation of infliximab) revealed no evidence of active or chronic injury in all segments that were sampled. The absence of features of chronicity on biopsies suggests that the colonic inflammation was more in keeping with a drug-induced acute event as opposed to preexisting inflammatory bowel disease exacerbated by the interleukin-17 monoclonal antibody. The patient received a total of 7 doses of infliximab, 3 during accelerated induction (weeks 0, 1 and 5) and every 4 weeks for 4 months. Unfortunately, the patients’ plaque psoriasis deteriorated clinically while on infliximab. In concert with the patients’ dermatologist, a decision was reached to bridge the patient onto an anti-IL12/23 neutralizing antibody with a phosphodiesterase type 4 (PDE4) inhibitor (4 week infliximab washout period). We anticipate that the use of ustekinumab will provide therapeutic coverage of the patient’s Crohns colitis and plaque psoriasis. Patient is presently asymptomatic from his IBD, CRP is 4 (< 8 mg/L) pending endoscopic reassessment. The patient will continue to be followed by Gastroenterology and Dermatology with monitoring of the patient’s clinical status, CRP and albumin.

## Discussion

This case describes a patient with a history of moderate plaque psoriasis, who developed severe Crohn’s-like colitis in the context of recent exposure to an IL-17 inhibitor. To our knowledge, this patient had no past history suggestive of CD affecting his gastrointestinal tract, nor any extra-intestinal manifestations. He does, however, have a number of risk factors for CD, including a first-degree family member with IBD, and a significant smoking history [15]. Furthermore, and perhaps most relevant to this particular case is that IBD is a well-established co-morbidity of psoriasis. A recent case-control study in Israel demonstrated that psoriasis patients were 2.5 times more likely to have CD and 1.6 times more likely to have UC [2]. Additionally, a large cohort study of women in the United States highlighted an increased risk of CD among those with psoriasis [3], while a nationwide cohort study in Denmark observed an increased risk of IBD associated with more severe presentations of psoriasis [4]. Ultimately, regardless of the patient’s exposure to Ixekizumab, evidence would suggest his baseline risk for manifesting CD was already increased compared to the general population.

Clinical trial data have proven Ixekizumab as an effective therapeutic agent for patients with moderate to severe psoriasis [10, 16–18]. The role of IL-17A in the inflammatory pathogenesis for psoriasis has thus been given credence through the clinical effect of its inhibition. Similarly, IL-17A release by Th17 cells has been implicated in the pathogenesis of IBD with serum, intestinal mucosal and fecal IL-17 levels having been shown to be increased in those with active CD [6, 7]. Nevertheless, as a therapeutic target in the treatment of IBD, the results of IL-17 inhibitors have not fared as well.

Functionally distinct T helper subtypes have unique cytokine profiles and roles in both health and disease. Th-17 cells predominantly produce IL-17 cytokine subsets to effect both protective and detrimental changes in tissue-specific immunity, particularly within mucosal surfaces and their interplay with extracellular and intracellular pathogens [19–25]. IL-23 induces Th-17 expansion, which increases IL-17 production, driving inflammation through IL-17A stimulated pro-inflammatory mediators released from leukocytes, keratinocytes and epithelial cells [22, 26, 27]. In psoriasis, IL-17A stimulated inflammation leads to acanthosis and plaque formation, which is reduced via it’s inhibition [27–29]. While in theory intestinal epithelial response to IL-17A is similar, multiple studies have highlighted the converse to be true. In mouse models, IL-17A or IL-17RA blockade resulted in colitis exacerbation [25, 26, 28, 30]. In one study, murine IL-17A/RA inhibition was associated with increased intestinal epithelial permeability, decreased antimicrobial peptide expression, reduced neutrophil aggregation and imbalance between regulatory and effector CD4+ T cells [28]. All of these factors lead to adverse bacterial translocation, inflammatory propagation and ultimate destruction of the epithelial barrier in mice [28]. These findings are consistent with previous studies associating IL-17 with a protective effect in T-cell driven intestinal inflammation, induction of protective intestinal epithelial gene expression, and increased mucosal defense against gut microbes [24–26, 28, 30–32].

In a proof-of-concept clinical trial of Secukinumab among a sample of 59 patients with moderate to severe CD, not only was IL-17 blockade ineffective, but was also associated with higher rates of adverse events and worsening of the disease itself [11]. A phase 2 clinical trial of Brodalumab in a similar population of CD patients was terminated early due to worsening CD symptoms in the treatment arm [12]. While the results of these trials are overwhelmingly negative, they may allude to a potential protective role of IL-17A in IBD. This is not a new hypothesis, and has been explored in a mouse model, where IL-17 was associated with a protective effect in T-cell driven intestinal inflammation [26].

Regarding Ixekizumab, there have been 2 recent post-hoc analyses looking at adverse events of suspected IBD among 4209 patients across 7 clinical for patients with moderate to severe psoriasis. Of the 19 IBD cases that were identified (CD, *N* = 7; UC, *N* = 12), 4 experienced flares, while the remaining 15 represented new cases (CD, *N* = 6; UC, *N* = 9) [12, 13]. The incidence rates of UC (1.08 per 1000 patient-years) and CD (1.85 per 1000 patient-years) were relatively consistent with occurrences of IBD across other trials of IL-17 inhibitors [5]. Nevertheless, a major limitation of these clinical trials was their data collection of IBD history, which was only recorded if volunteered by the participants. Therefore, the number of patients with a history of IBD may very well be underreported. Furthermore, two patients had CD-related adverse events during the induction period, after 64 and 88 days of exposure [13, 14].

This is relatively similar to the present case, where symptoms started after 86 days of exposure, approximately 2 days after completing the 84-day induction period. Overall, both of these studies concluded that the IBD incidence rates in patients treated with Ixekizumab was comparable with those in observational studies of psoriasis populations.

A recent review by Hohenberger speaks to the association of IL-17 inhibition with colitis exacerbation, and collating murine evidence with clinical trial data, IL-17A inhibition in the setting of intestinal inflammation further exacerbates disease [29]. It can further be hypothesized that certain patient populations, with the unfortunate combination of genetic pre-disposition and microbiome pathogenicity, may be at risk for developing de novo inflammatory bowel disease in the setting of IL-17A inhibition.

To date, there have been no case reports of Crohn’s disease manifesting among psoriasis patients treated with Ixekizumab. A case report was published in 2018 involving a 31 year old male with plaque psoriasis who developed similar symptoms after being treated with Ixekizumab for 3 months [33]. Flexible sigmoidoscopy revealed absent vascular pattern and severe ulceration, biopsies revealed chronic lymphoplasmacytic infiltration confirming a diagnosis of ulcerative colitis. The patient was unresponsive to steroids but did respond to anti-TNF therapy. A similar report was published in 2017 involving a 53-year-old male with refractory psoriasis who experienced analogous symptoms of fever, abdominal pain and oral ulcers after 8 weeks of exposure to Secukinumab [34]. Colonoscopy and gastroscopy revealed only mild ulceration, with non-specific biopsies for inflammatory cellular infiltrates. The patient was diagnosed with CD/Behcet’s disease, and responded well to 40 mg per day of oral prednisone. Fortunately, following discontinuation of the biologic, the patient did not have any relapses or findings on repeat colonoscopy. Our patient, in comparison, manifested a more severe presentation with diffuse colonic involvement requiring a prolonged course of steroids with induction utilizing escalated anti-TNF therapy. Unlike the other cases, our patient’s plaque psoriasis flared while on anti-TNF requiring a transition onto anti-IL 12/23 therapy which is currently maintaining both his CD and plaque psoriasis.

In conclusion, we present a case of Crohn’s-like colitis in a 43-year-old male who had recently undergone induction with an IL-17 inhibitor, Ixekizumab, for treatment of refractory plaque psoriasis. Although this was a first presentation of colitis in this patient, he had known risk factors including a first-degree family member with IBD, a 20 pack-year smoking history, and moderate to severe psoriasis. Although recent clinical trials of Ixekizumab have revealed a small cluster of new IBD cases among psoriasis patients, the incidence rates do not appear to be different compared to those rates seen in the general psoriasis population. Ultimately, there is still a dearth of evidence to suggest that this particular biologic is complicit in this patient’s new diagnosis of Crohn’s disease. Until more complete longitudinal safety data is available, it would be prudent to closely monitor these patients when prescribing IL-17 inhibitors, especially in those with other risk factors for IBD. A biomarker such as fecal calprotectin would be ideally suited for this purpose [35].

## Data Availability

N/A

## Abbreviations

CD: Crohn’s disease
CMV: Cytolomegalovirus
CRP: C-reactive protein
ED: Emergency Department
IBD: Inflammatory bowel disease
IL: Interleukin
PDE4: Phosphodiesterase type 4
TNF: Tumor necrosis factor
UC: Ulcerative colitis
